# Ag nanoparticle decorated MnO_2_ flakes as flexible SERS substrates for rhodamine 6G detection†

**DOI:** 10.1039/c8ra07778a

**Published:** 2018-11-09

**Authors:** Yan Zhang, Rui Jia Liu, Xiaofei Ma, Xiao Ying Liu, Yu Xin Zhang, Jie Zhang

**Affiliations:** State Key Laboratory of Mechanical Transmissions, College of Materials Science and Engineering, Chongqing University Chongqing 400044 P. R. China zhangyuxin@cqu.edu.cn; The Key Laboratory of Optoelectronic Technology & System, Education Ministry of China, Chongqing University Chongqing 400044 P. R. China zhangjie@cqu.edu.cn; China Academy of Space Technology (Xi'an) Xi'an 710000 P. R. China; Engineering Research Center for Waste Oil Recovery Technology and Equipment of Ministry of Education, College of Environment and Resources, Chongqing Technology and Business University Chongqing 400067 China

## Abstract

Smart design of advanced substrates for surface-enhanced Raman scattering (SERS) activity is challenging but vital. Herein, we synthesized a new kind of AgNPs/MnO_2_@Al flexible substrate as a SERS substrate for the detection of the analyte rhodamine 6G (R6G). The fabrication of porous MnO_2_ nanoflakes on Al foil was conducted *via* a facile hydrothermal strategy. Owing to the large active surface area of the MnO_2_ nanoflakes, the Ag nanoparticles were immobilized and displayed superior SERS performance with a low detection concentration of 1 × 10^−6^ M for R6G. In addition, the SERS performance was found to be strongly related to the morphology of the MnO_2_@Al substrate material. Our smart design may provide a new method of construction for other advanced SERS substrates for the detection of R6G.

## Abstract 2

In this work, we synthesized a new kind of AgNPs/MnO_2_@Al flexible substrate as a SERS substrate for the detection of analyte Rhodamine 6G (R6G), which displayed superior SERS performance with low detection concentration of 1 × 10^–6^ M for R6G.

## Graphical abstract

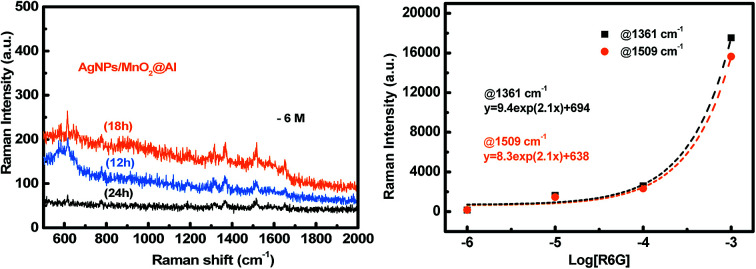

## 1. Introduction

Surface-enhanced Raman spectroscopy (SERS) is a powerful and extremely sensitive analytical technique, which has attracted wide attention since it was first used to observe a rough Ag electrode.^1^ Until now, it has been intensely explored for use in many areas including biodetection, biomedicine, environmental monitoring, analytical chemistry, and so forth.^2–6^ Recently semiconductor nanostructures have been found to show the potential for SERS enhancement.^7,8^ Many researchers have proved that synthesized semiconductor–noble metal nanocomposites possess obvious Raman enhancement effects. For example, Zhao *et al.* fabricated a MoS_2_ nanosheet-silver nanoparticles composite for the detection of 2-mercaptobenzimidazole (MBI) molecules.^9^ Yang *et al.* synthesized an Ag-coated Fe_3_O_4_ microsphere using the solid-phase method, which was used to detect 4-aminothiophenol with a low concentration of 1.0 × 10^−12^ M.^10^ In addition, a noticeable increase in the Raman activities of molecules adsorbed onto Au–SiO_2_,^11,12^ Au–TiO_2_,^13^ Ag–CuO^14^ Ag-carbon,^15^ has also been reported. All of these works indicate that the supporting substrates (metal oxides (MOx)/nanoparticles (NPs)) have a synergistic effect on the properties of the intrinsic NPs, giving the composites a much greater potential for use in SERS applications compared to the NPs alone.^16–18^ The properties of MOx can be modified by incorporating metal NPs and thus obtaining a great enhancement in the SERS signals.^19,20^ Moreover, several studies have focused on fabricating flexible SERS substrates, in order to further develop their innovative use as bendable supports. Compared with conventional rigid substrates, flexible substrates endow opto-electronic devices with unique properties, such as flexibility, portability and/or disposability. Fox example, Pimentel *et al.*^21^ fabricated a ZnO nanorod decorated with Ag nanoparticles supported by a flexible cardboard platform. When using rhodamine 6G (R6G) as the test analyte, a Raman enhancement factor of 7 × 10^5^ was obtained for this SERS substrate, and the distribution of “hot spots” on the substrate is generally believed to affect the SERS performance.^22^ Later, Oliveira *et al.*^23^ distributed Ag nanostars on a paper with high porosity, and this exhibited a better SERS performance than that of the paper with low porosity. More recently, a new substrate composed of Ag nanoflowers on a graphene@Cu net (AgNFs/G@Cu) was prepared by Zhang *et al.*,^24^ who obtained a flexible substrate for flexible SERS application. These works suggest that porous, flexible and conductive substrates show efficient SERS enhancement.

As a common metal oxide, MnO_2_ has been extensively investigated owing to its low cost, natural abundance, various morphologies, and low toxicity.^25^ These advantages provide it with a wide range of applications in catalysis, ion exchange, molecular adsorption, biosensors, and energy storage.^26^ MnO_2_ has various constructions including particles, rods, wires, tubes, sheets and 3D porous nanostructures, in which porous MnO_2_ frameworks are a significant research area owing to their superiority to bulk counterparts in many aspects, such as their higher surface area and the fact that they possess a lower dead volume.^27^ This provoked us to develop novel composite materials as highly SERS-active substrates.

Herein, we report a facile method to fabricate a flexible MnO_2_ porous construction on aluminum foil using a hydrothermal method, and subsequently decorate these porous flakes structures with Ag nanoparticles (AgNPs) achieved using a wet chemical method. Three kinds of MnO_2_@Al supports with different frameworks were obtained by controlling the hydrothermal reaction time of the Al foil in the KMnO_4_ solution. During the following investigation, we found that the loading amount of the AgNPs is dependent on the morphology and structure of the MnO_2_@Al substrate. By comparing the SERS performance of the three kinds of support, the AgNPs/MnO_2_@Al (18 h) sample was found to have the best properties for the detection of R6G. This is attributed to the 3D porous framework which offers more sites for aggregation of the Ag particles, giving the SERS signal enhancement. In addition, we found a new application for Al foil in this study, which could be helpful for further investigation of other metal foils.

## 2. Experimental section

### 2.1 Materials and substrates

Commercially available Al foil (99.9% purity) was obtained from Sino pharm Chemical Reagent Ltd. Co. All other reagents and solvents were purchased from Alfa Aesar and were analytical grade and used without further purification. High-purity Milli-Q water (18.2 MΩ) was used throughout the study.

### 2.2 Preparation of MnO_2_ nanosheets on Al foil

Aluminum foil (30 × 30 mm) was first immersed in the diluted hydrochloric acid solution to remove the oxide/hydroxide layer from the surface, and cleaned with acetone, alcohol and deionized water *via* ultrasonication for 10 min to remove the organic pollutants respectively. After drying with high purity N_2_, it was immersed in a solution containing KMnO_4_ (0.1 M, 30 mL). The aqueous solution and the Al foil were transferred to a Teflon-lined stainless-steel autoclave, which was maintained at 160 °C for 12, 18 and 24 h, respectively. Subsequently, the sample was washed with distilled water and ethanol, and dried at 60 °C overnight.

### 2.3 Preparation of AgNPs/MnO_2_@Al nanocomposites for SERS measurement

An AgNPs/MnO_2_@Al hybrid was synthesized *via* a wet chemical method. Briefly, silver sol was first prepared by dropping trisodium citrate (0.34 mM, 10 mL) into the silver nitrate solution (90 mg, 500 mL) which was boiling and being stirred. After cooling to room temperature, the obtained silver sol was aged for several days before use. During the heating process, the color of the mixture changed from colorless, to yellow, then orange and then finally a grayish yellow. Moreover, an obvious black precipitate was generated at the bottom of the beaker. At last, different MnO_2_@Al samples were immersed in the silver sol solution for 0.5 h and dried in air.

### 2.4 Characterization and SERS measurement

Powder X-ray diffraction (XRD, D/max 2500, Cu Kα) was used to identify the composition of the samples. The surface morphologies of the samples were observed using scanning electron microscopy (SEM) (ZEISS AURIGA FIB/SEM) with an energy dispersion X-ray spectrometer (EDS). Atomic force microscopy (AFM) (Nanonavi E-Sweep, SII, Japan) was also used in the tapping mode at room temperature in air. R6G solutions (2 μL) (Sigma-Aldrich) of different concentrations were dropped onto samples before the SERS measurement. The SERS spectra were acquired with a laser confocal Raman spectrometer (Horiba Jobin Yvon LabRAM HR Evolution) equipped with a 50× objective lens of numerical aperture (NA) 0.75, at a work distance (WD) of 0.37 mm, using an air cooled frequency doubled Nd:YAG green laser (*λ* = 633 nm, *P* = 50 mW with a 10% filter) at room temperature. An accumulation time of 2 s was used in the tests to avoid the heating effect generated by the laser.

## 3. Results and discussion

### 3.1 Synthesis and characterizations of AgNPs/MnO_2_@Al

**Scheme 1:**
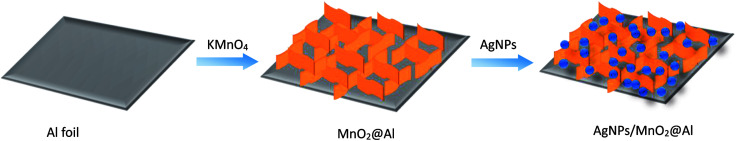
Schematic illustration of the synthesis process for the AgNPs/MnO_2_@Al array.

Scheme 1 depicts the synthesis route for the AgNPs/MnO_2_@Al. During the process, the MnO_2_ nanoflakes were decorated onto the surface of the Al foil *via* a reaction with the KMnO_4_ solution under hydrothermal conditions. The reaction involved in the formation of the MnO_2_ nanosheets is expressed as follows:^28^

1 2KMnO_4_ + 2H_2_O → 2MnO_2_ + 2K^+^ + 4OH^−^ + O_2_

Impressively, the MnO_2_ sheets provide abundant immobilization sites for the silver nuclei, as well as a spacious template for the adhesion of the silver nanoparticles.

The silver colloid was prepared *via* the chemical reduction method and based on the research published by Lee and Meisel. During the process, silver nitrate serves as the silver precursor and trisodium citrate is the reductant.^29^ The reaction mechanism can be expressed as follows:^30^

2 4Ag^+^ + C_6_H_5_O_7_Na_3_ + 2H_2_O → 4Ag^0^ + C_6_H_5_O_7_H_3_ + 3Na^+^ + H^+^ + O_2_

**Fig. 1:**
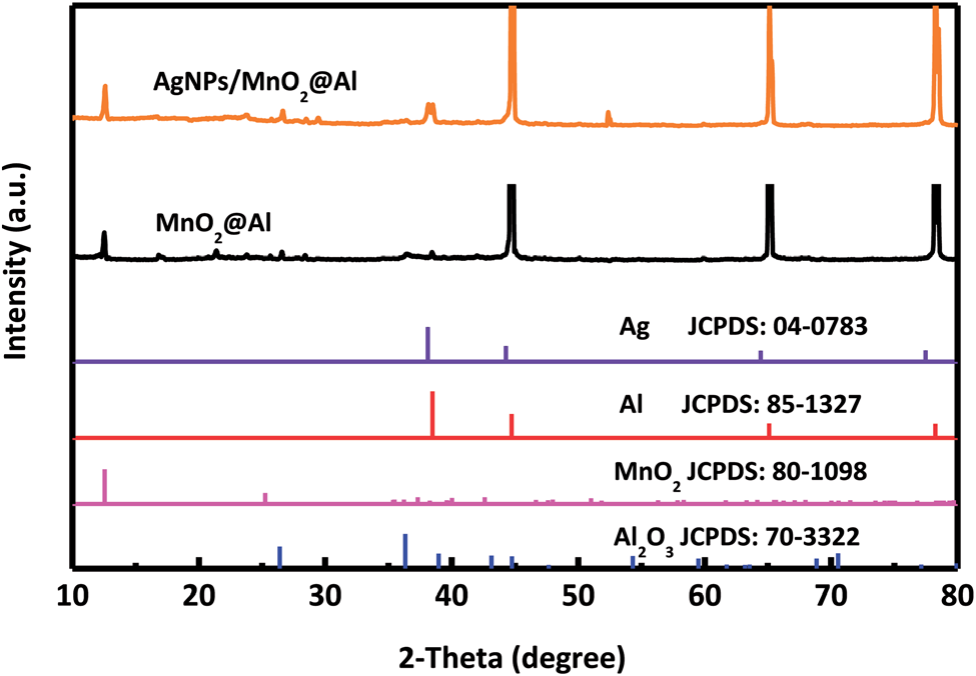
XRD pattern of MnO_2_@Al and AgNPs/MnO_2_@Al composite.

The single crystalline characteristics of MnO_2_@Al and AgNPs/MnO_2_@Al were investigated using XRD, and the corresponding spectra are shown in Fig. 1. For these two samples, the diffraction peaks at about 12.5°, 36.2° and 42.5° from the MnO_2_ match the standard XRD pattern for a birnessite-type manganese oxide crystal (JCPDS 80-1098).^31^ Although the standard XRD pattern for Ag (JCPDS 04-0783)^32^ and Al (JCPDS 85-1327) are similar, a new peak for a (111) plane can be clearly observed for the AgNPs/MnO_2_@Al sample, but not for the MnO_2_@Al sample, suggesting the successfully fabrication of the AgNPs on the MnO_2_@Al substrate. In addition, some undefined peaks corresponding to the Al_2_O_3_ phase (JCPDS 70-3322) are due to the strong oxidation ability of KMnO_4_.

**Fig. 2:**
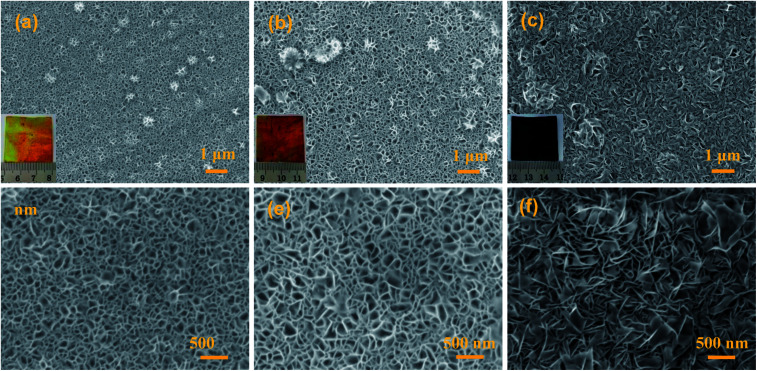
The SEM images of MnO_2_@Al prepared with different lengths of time: (a and d) 12 h; (b and e) 18 h; (c and f) 24 h, the inset is the digital image of the corresponding products.

Fig. 2 presents typical SEM images and photos for the MnO_2_@Al products obtained at different reaction times. As shown in Fig. S1 (ESI†), the surface of the original aluminum foil is smooth and shows a silver color in the inset of Fig. S1.† After the aluminum foil reacts with the KMnO_4_ solution at 160 °C for different times, MnO_2_@Al samples with various porous frameworks consisting of MnO_2_ nanoflakes are clearly observed in Fig. 2a–c. Meanwhile, the color gradually changes from a brass color to black as seen in the insets of Fig. 2a–c. After 12 h under hydrothermal conditions, the net-like morphology of the MnO_2_ nanosheets was formed on the aluminum foil (Fig. 2a and d). When the reaction process was increased to 18 h, the porous structure was found to be larger than that after 12 h (Fig. 2b and e). However, the MnO_2_ nanoflakes tended to aggregate and became crossed with each other as the time was extended to 24 h (Fig. 2c and f), which would thus reduce the porous structure. In addition, Fig. S2 (ESI†) shows a cross-section SEM image for the MnO_2_@Al prepared at 18 h, which also suggested that the net-like framework was made of flakes.

**Fig. 3:**
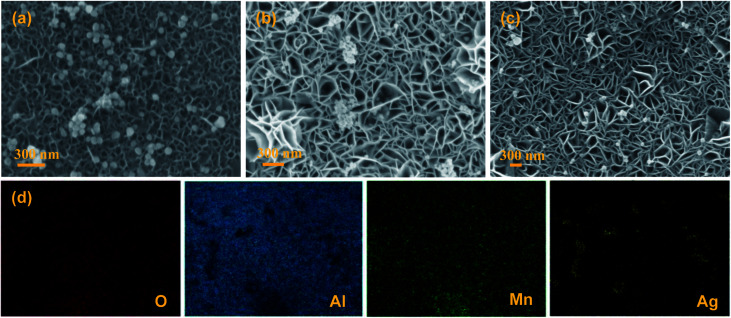
The SEM images of (a) AgNPs/MnO_2_@Al (12 h); (b) AgNPs/MnO_2_@Al (18 h); and (c) AgNPs/MnO_2_@Al (24 h). (d) The corresponding EDS mapping for AgNPs/MnO_2_@Al (18 h).

The SEM images of the AgNPs/MnO_2_@Al samples are presented in Fig. 3. It was observed that the AgNPs were distributed on the top surface of the MnO_2_ nanoflakes when the MnO_2_@Al (12 h) acts as the support (Fig. 3a). However, except for the AgNPs scattered on the outside surface of the MnO_2_@Al (18 h) substrate, many of the silver particles embedded in the porous framework were made of larger MnO_2_ nanosheets (Fig. 3b). As the hydrothermal time was increased to 24 h, the AgNPs were found to be distributed on the surface of porous structure again, rather than embedded in the pores (Fig. 3c), which was due to the crossed over larger MnO_2_ sheets which narrow the holes. The element mapping images of the AgNPs/MnO_2_@Al sample (18 h) are shown in Fig. 3d. It can be seen that there is a highly homogeneous distribution of the O, Al and Mn in the sample, which is in line with the results from SEM obtained previously. With respect to the element distribution of Ag, the silver particles aggregated on the surface of the MnO_2_ flakes can be clearly observed, while those embedded in the porous framework are not included.

**Fig. 4:**
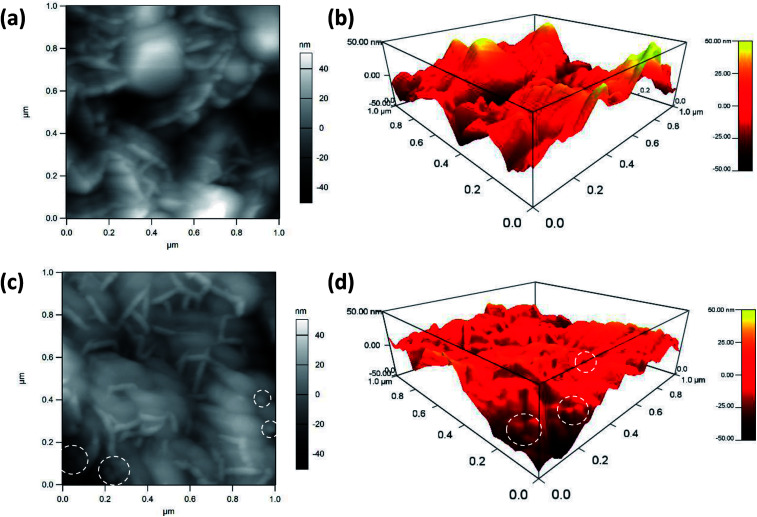
AFM images of MnO_2_@Al (18 h) before (a), (b) and after (c), (d) deposition of silver colloidal nanoparticles. The white dotted circles show the location of the AgNPs on the MnO_2_@Al substrate.

Atomic force microscopy is a highly precise technique that can provide topographical and compositional information on the surface of materials. Herein, AFM pictures of the MnO_2_@Al (18 h) substrates before and after the deposition of silver nanoparticles are shown in Fig. 4. The porous structure can be observed from the Fig. 4a and b, which is in accordance with results previously obtained using SEM. After the deposition of silver nanoparticles, the nanoparticles can clearly be seen from the AFM picture (the white dotted circles in Fig. 4c and d), confirming that the silver particles are embedded into the MnO_2_ flakes successfully. In addition, the AgNPs are randomly distributed on the surface and are prone to aggregation during the deposition process. Analysis of the AFM data (Fig. 4b and d) indicates that the AgNPs/MnO_2_@Al (18 h) show a greater nanoscale roughness (RMS = 14.75 nm) compared to the hierarchical MnO_2_@Al (18 h) architecture (RMS = 9.69 nm), which may be ascribed to more Ag NPs being embedded in the holes resulting in the reduction of pores.^33,34^

### 3.2 Raman spectrum

**Fig. 5:**
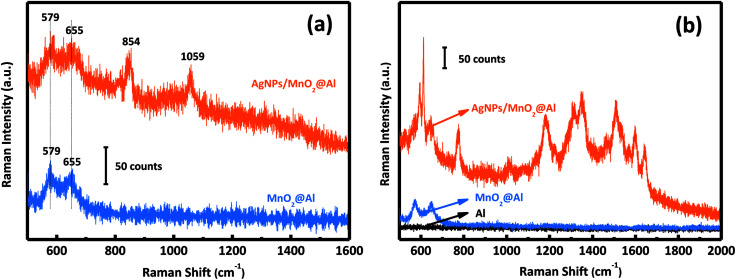
(a) Raman spectra of MnO_2_@Al and AgNPs/MnO_2_@Al; and (b) SERS activity of Al foil, MnO_2_@Al, and the AgNPs/MnO_2_@Al composite for R6G.

Fig. 5a shows the Raman spectra of the MnO_2_@Al and AgNPs/MnO_2_@Al samples. The AgNPs/MnO_2_@Al product shows two additional Raman peaks at about 854 and 1059 cm^−1^, the peak at 1059 cm^−1^ belongs to the aromatic C–H bending of the silver particles, while 854 cm^−1^ is not a typical peak and may be caused by complex coupling among MnO_2_, Al and Ag.^35^

### 3.3 SERS activity of Al foil, MnO_2_@Al and AgNPs/MnO_2_@Al

The SERS activities of these three samples were evaluated using the detection of R6G. Fig. 5b depicts the SERS spectra of R6G on the AgNPs/MnO_2_@Al composite (top curve), MnO_2_@Al (middle curve) and Al foil (bottom curve). There is no obvious SERS signal for the Al foil and MnO_2_@Al samples, but AgNPs/MnO_2_@Al shows a prominent improvement (at 613, 1181 and 1506 cm^−1^) for the detection of R6G. By comparison, it is clear that the role of the AgNPs in this configuration is the key to the detection of the R6G analyte.^36^ Moreover, the high density of AgNPs results from the large specific surface area and the porous structure of the immobilized MnO_2_ flakes can generate more hot spots and increase the loading of the analyte, which is beneficial to the SERS signal.^9,37^

**Fig. 6:**
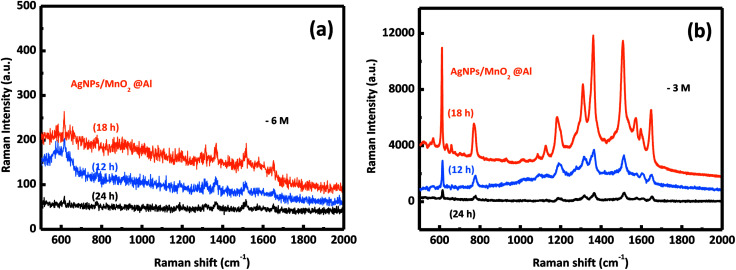
The SERS spectra of the R6G molecules collected from the AgNPs/MnO_2_@Al samples (12 h, 18 h, 24 h) at different concentrations of (a) 10^−6^ M and (b) 10^−3^ M.

In our previous report, the amount of AgNPs played an important role in the detection of R6G. In this work, it was also found that the amount of adhesion of the AgNPs was also strongly dependent on the structure and morphology of the substrate. We systematically investigated the influence on the detection of R6G through a series of experiments comparing the three kinds of MnO_2_@Al supports while keeping the other conditions unchanged. The SERS spectra for 10^−3^ M and 10^−6^ M of R6G molecules adsorbed onto the samples AgNPs/MnO_2_@Al (12 h), AgNPs/MnO_2_@Al (18 h) and AgNPs/MnO_2_@Al (24 h) are presented in Fig. 6a and b respectively. In a comparison to the SERS signals for the three substrates, the SERS intensity decreased in the sequence of AgNPs/MnO_2_@Al (18 h), AgNPs/MnO_2_@Al (12 h) and AgNPs/MnO_2_@Al (24 h). When using AgNPs/MnO_2_@Al (18 h), the SERS intensity of R6G increased almost four times for the 1361 cm^−1^ peak compared with the AgNPs/MnO_2_@Al (24 h) sample. The enhanced outcomes are due to the high density of the AgNPs and small “hot spots” on the SERS substrates, which causes a strong plasmonic coupling effect between the adjacent AgNPs,^38^ and where the electromagnetic field can be amplified dramatically.^39^ For the sample AgNPs/MnO_2_@Al (18 h), more AgNPs were adsorbed onto the porous framework made of MnO_2_ sheets, in other words, the gap between the two adjacent AgNPs decreases as the silver loading increases, which can be confirmed using the SEM images.

**Fig. 7:**
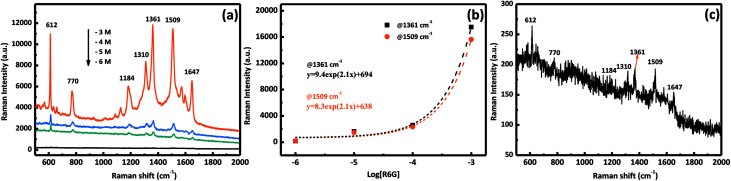
(a) SERS spectra of R6G with six different molecular concentrations of AgNPs/MnO_2_@Al (18 h); (b) SERS intensity at 1361 cm^−1^ (squares) and 1509 cm^−1^ (circles) for different concentrations of R6G, the curves represent the fit to the experimental data; and (c) the SERS spectrum of R6G at 10^−6^ M.

To demonstrate the performance of our prepared AgNPs/MnO_2_@Al (18 h), the SERS sensitivity was investigated with different concentrations of the R6G solution ranging from 10^−6^ M to 10^−3^ M. As shown in Fig. 7a, the SERS detections were completed by altering the concentration of R6G. Clear peaks were observed in the three curves, and the vibration peaks at 1184, 1310, 1361, 1509, and 1647 cm^−1^ were assigned to the C–O–C stretching, C–H in-plane bending, and C–C stretching of the aromatic ring of the R6G molecule, respectively, while the peak at 770 cm^−1^ was derived from the hydrogen atoms of the xanthene skeleton and the out-of-plane bending motion.^37,40,41^Fig. 7b shows the relationship between the SERS intensity of R6G and its concentration at 1361 cm^−1^ and 1509 cm^−1^, which is an exponential increase. The Raman signal is still observable when the molecule concentration is as low as 10^−6^ M (Fig. 7c), in which the relative intensities of 612 and 1509 cm^−1^ are about 50 and 40 counts, respectively, indicating the high sensitivity of our substrates. The excellent performance can be ascribed to the porous structure of the MnO_2_ sheets which provides more sites for aggregation of Ag particles.

## 4. Conclusions

In conclusion, we have used a facile hydrothermal and wet chemical method to construct flexible substrates based on incorporating AgNPs onto MnO_2_ nanoflakes supported on Al foil. The as-prepared AgNPs/MnO_2_@Al flexible sample was characterized in detail using instrumental techniques such as SEM, XRD and AFM. Owing to their porous, flexible and conductive features, a high sensitivity (10^−6^ M) was obtained when using the AgNPs/MnO_2_@Al as a SERS substrate for the detection of R6G. Moreover, the effect of the MnO_2_ morphology on the SERS performance were discussed. The experiment results suggest that the AgNFs/MnO_2_@Al substrates could be used for novel and practical SERS applications in the dyestuff industry.

## Conflicts of interest

There are no conflicts to declare.

## Supplementary Material

RA-008-C8RA07778A-s001
