# Supplementary material for: Ag nanoparticle decorated MnO2 flakes as flexible SERS substrates for rhodamine 6G detection
Source: RSC Adv. 2018 Nov 9;8(66):37750–6. doi: 10.1039/c8ra07778a (PMC9089333; doi:10.1039/c8ra07778a)

Electronic Supplementary Material (ESI) for RSC Advances.
This journal is © The Royal Society of Chemistry 2018

Supplementary Information

Fig. S1 The SEM images of Al foil, the insert is the digital image.

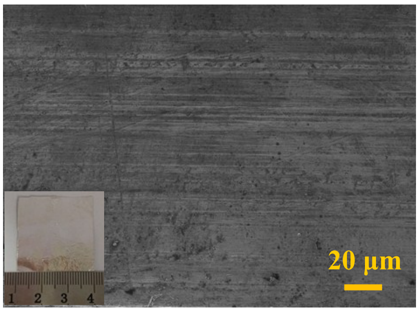

Fig. S2 The Cross-section SEM image of the Al@MnO_2 prepared at 18 h.

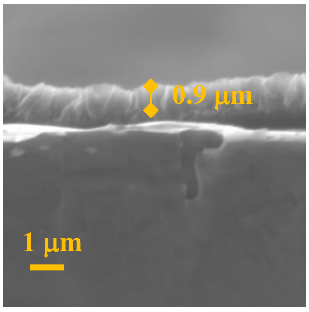

Supplement: RA-008-C8RA07778A-s001 [file RA-008-C8RA07778A-s001.pdf]
